# Under Pressure: Interactions between Commensal Microbiota and the Teleost Immune System

**DOI:** 10.3389/fimmu.2017.00559

**Published:** 2017-05-15

**Authors:** Cecelia Kelly, Irene Salinas

**Affiliations:** ^1^Center for Evolutionary and Theoretical Immunology, Department of Biology, University of New Mexico, Albuquerque, NM, USA

**Keywords:** microbiota, commensals, teleost, fish, immunity, mucosal immunity, evolution

## Abstract

Commensal microorganisms inhabit every mucosal surface of teleost fish. At these surfaces, microorganisms directly and indirectly shape the teleost immune system. This review provides a comprehensive overview of how the microbiota and microbiota-derived products influence both the mucosal and systemic immune system of fish. The cross talk between the microbiota and the teleost immune system shifts significantly under stress or disease scenarios rendering commensals into opportunists or pathogens. Lessons learnt from germ-free fish models as well as from oral administration of live probiotics to fish highlight the vast impact that microbiota have on immune development, antibody production, mucosal homeostasis, and resistance to stress. Future studies should dissect the specific mechanisms by which different members of the fish microbiota and the metabolites they produce interact with pathogens, with other commensals, and with the teleost immune system.

## Introduction

Teleost fish are colonized soon after hatching by a diverse set of microbes, which interact with, and shape the development of the host immune system. Like mammals, teleosts have mucosal surfaces, which serve as the first line of defense against invading pathogens, but also harbor non-pathogenic microbes, which makeup the host microbiome. Microbiome studies in a variety of plant and animal species have been accomplished in recent years using rapidly advancing sequencing technologies. These studies continue to expand the knowledge base needed to implement microbiome manipulations with the goal of improving host health. Understanding microbiota-immune system interactions in teleosts is important both for developing solutions to aquacultural problems and for further refinement of fish models, such as the zebrafish (*Danio rerio*), as useful models for biomedical research.

Studies on a number of metazoan hosts have shown that the composition of the microbiota does not merely reflect that of the environment but rather a specific selection of microbial assemblages by hosts has occurred over time (1). Several studies have already determined the bacterial community composition at different teleost mucosal sites (2–5), as well as the presence of a core microbiome in the gut of zebrafish (6). Unfortunately, studies pertaining mycobiomes and viriomes of fish are lacking. As a consequence, this review only discusses interactions between bacteria and fish immune systems.

As discussed throughout this review, microbiota exert direct effects on the teleost immune system through their display of microbe-associated molecular patterns (MAMPs) and secretion of factors. Microbiota and their secreted molecules can act locally on the mucosal epithelium or systemically if they enter host circulation or activate immune cells that then travel from mucosal sites to systemic lymphoid tissues. Additionally, these microbes can exert immunostimulatory or immunosuppressive effects on both innate and adaptive immune cells, specific examples of which will be discussed further in this review.

One of the most intimate relationships between microbiota and vertebrate mucosal immune systems is the coevolution between microorganisms and mucosal antibodies (7). Thus, in this review, we will describe in detail, current findings regarding how microbiota shape teleost B cell and antibody responses and how mucosal antibodies and secretory component (SC) allow the host to sculpt its microbial communities. Despite immune exclusion mechanisms present in teleosts, it is clear that certain microbes are capable of reaching and occupying the epithelium of teleosts (2, 8). Similar to mammals, microbial populations vary greatly over the various mucosal body sites of a single fish, with the biggest differences seen between GI tract and external mucosal surfaces (i.e., skin, nose, and gill) bacterial communities (2), suggesting unique and specialized symbiotic relationships at each mucosal site. Conversely, microbial species specific to different mucosal sites may have coevolved alongside the host to perform essential physiological or metabolic duties critical for the optimal functioning of each mucosal immune compartment.

Following the identification of whole microbiome compositions in various fish species, several groups have gained ground in identifying specific microbial species, which are capable of modulating the immune system by colonizing germ-free fish with a single microbe (monocolonization) or a defined group of microbes. These studies have primarily been accomplished using zebrafish (*Danio rerio*) a model for which good germ-free rearing techniques were developed in 2004. Zebrafish are a small, genetically manipulable, and provide the advantage of being transparent during the larval life stage, which makes them a useful model for studying immune system dynamics in response to microbial colonization. Only recently, germ-free seabass (*Dicentrarchus labrax*) have been produced, allowing the study of the interactions between microbiota and a fish host in the seawater environment (9). Future work focused on the identification of candidate microbial species, which can be introduced *via* probiotics or eliminated using antibiotics will be essential to produce treatment plans applicable to improving fish health in aquaculture conditions.

In this review, we will focus on the role of the microbiota in the development and function of the teleost immune system. We will discuss mucosal immune responses at the various tissues that harbor these microbial communities, as well as systemic immune responses, which are regulated by microbiota and their products. We will also review recent studies, which have shed more light on the abilities of individual microbial species to influence the teleost immune system or provide protection from pathogens. Last, we also aim to synthesize known information and create a big picture model showing the different ways microbes and microbial products influence teleost immunity. This model takes into consideration the influence of the environment as well as other factors that can break the equilibrium between the microbiota and the fish host.

## The Immune System of Teleost Fish

The immune system of teleost has been studied for decades. Teleost fish have an immune system that resembles that of other jawed vertebrates. The teleost innate immune system provides a first line of defense by detecting and eliminating invading pathogens in an immediate and non-specific manner. Teleost fish also have an adaptive immune system, which relies on somatic recombination of germline-encoded V-D-J fragments to generate a vast repertoire of antigen receptors expressed on the membrane of T and B lymphocytes.

Due to the large number and diversity of teleost species (>30,000), we find unique evolutionary innovations in certain clades. At times, these innovations challenge the current dogma of mammalian immune systems. For instance, the Gadoid family lacks MHC-II expression and CD4 T cell-related molecules. Thus, this teleost group does not rely on traditional antigen presentation *via* the MHC-II and activation of T helper cells to mount adaptive immune responses and instead displays an expansion in the number of MHC-I genes (10, 11).

With regards to the anatomical organization of the teleost immune system, teleosts possess both primary and secondary lymphoid tissues. Primary lymphoid tissues include the thymus, where T cell development occurs, and the head-kidney, which performs hematopoietic functions similar to the mammalian bone marrow. Secondary lymphoid tissues include the spleen and the mucosa-associated lymphoid tissues (MALTs).

Teleost fish have four MALT, the gut-associated lymphoid tissue (GALT), the gill-associated lymphoid tissue (GIALT), the skin-associated lymphoid tissue (SALT), and the nasopharyx-associated lymphoid tissue (NALT) (12). These four MALT share important canonical features that underscore the conserved mechanisms of mucosal immunity in teleost fish (13, 14). Due to the important and direct interactions between commensals and teleost mucosal surfaces, we will describe in further details the organization and functioning of teleost MALT and their components in this review.

A continuously produced mucus layer covers the intestinal, gill, skin, and nasal mucosal surfaces of fish. The teleost mucus layer contains molecules with immunologically important properties, which interact directly with commensal microbial populations at mucosal surfaces. Thus, the composition of the teleost commensal bacteria, fungal, and viral communities is likely shaped by the physicochemical properties of the mucosal secretions. Currently, how the microbiota modulates the amount of mucus secretion as well as the specific composition of the secretions in teleosts is not well understood. While we know that mucosal infections in teleosts can alter the amount of mucus produced as well as the glycosylation levels of mucins (15, 16), how these changes alter the microbiome requires careful investigation.

Generally speaking, teleost MALTs do not contain organized lymphoid structures such as those found in endotherms. Thus, teleost MALTs are composed of a diffuse network of myeloid and lymphoid cells. However, within the GALT, there are some accumulations of T lymphocytes known as the interbranchial lymphoid tissue (ILT) (17). Although this structure does not present fully organized B and T cell regions and lacks germinal centers, it represents and ancient example of lymphocytic groupings at mucosal surfaces.

In mammals, the microbiota plays a pivotal role in the education of local antigen-presenting cells. The mechanisms of antigen uptake and antigen presentation in teleost MALT are not as well defined as those present in mammalian MALT, but it is clear that teleost MALT have significant numbers of antigen-presenting cells at mucosal sites. Dendritic cells (DCs), macrophages, IgT/Z^+^ B cells, and granulocytes have all been described to uptake antigen in teleost MALT (12, 18, 19). Additionally, enterocytes can uptake antigens by endocytosis (18). Finally, putative M-like cells have been described in the gut of rainbow trout (20). In mammals, luminal sampling DCs can directly sample symbiotic bacteria and transport them to draining lymph nodes (21). Importantly, the presence of the microbiota is required for the establishment of a tolerogenic phenotype in mucosal APCs. To date, the interactions between the microbiota and mucosal APCs of fish have not been investigated.

T cells are the most abundant of all the immune cells present in the MALT of teleost fish. Mucosal T cells include both CD8^+^ and CD4^+^ T cells. Recent reports in zebrafish and trout have shown that CD4^+^ T cells account for 10 and 20% of all T cells in gills and gut (22). However, phenotypic and functional studies on teleost mucosal CD4^+^ T cells are still lacking. CD8^+^ T cells are also present in GALT, GIALT, SALT, and NALT (23–25). Mucosal CD8^+^ T cells appear to have a cytotoxic (CTL) phenotype (12, 23, 25). Compared to systemic CD8α T cells, mucosal CD8α T cells also display markers characteristic of mammalian tissue resident memory T cells. Importantly, each teleost MALT harbors unique CD8α T cell subpopulations, as evidenced by the unique expression of adhesion molecules and receptors in NALT- and GALT-sorted CD8α T cells. Additionally, trout NALT contains two different populations of CD8α T cells located in the apical mucosal epithelium and the lateral neuroepithelium, respectively (25). Whether other teleost MALT harbor unique tissue microenvironments containing unique T cell subsets is unknown.

B cells are also part of all teleost MALT and have been fairly well characterized in all four MALT of rainbow trout (13, 14, 26, 27). In sharp contrast to the distribution of B cells in systemic lymphoid tissues, teleost MALT consistently contains a 50/50% distribution of IgM^+^ and IgT^+^ B cells (13, 14, 26, 27). The discovery of IgT as the chief mucosal Ig in teleosts opened up a number of questions regarding the role of this molecule in the maintenance of symbiotic communities in teleost fish. As discussed later, mucosal IgT responses take place in a compartmentalized manner in response to mucosal pathogens. Importantly, commensal bacteria modulate B cells and mucosal Igs.

## The Teleost Fish Microbiome

Although the presence of microbial communities on the mucosal surfaces of teleost fish has been acknowledged for decades, the composition, topography, and environmental factors that shape teleost bacterial microbiomes have only recently been unveiled thanks to deep sequencing of the 16S rDNA variable region.

Currently, most of the research efforts, which aim to understand the fish microbiota have focused on sequencing bacterial communities from aquacultured species. Microbiome studies from wild fish are also available (28) but less well studied (3, 29). Since phylogeny is a determining factor of the microbial composition of the host (1), and given the large number and taxonomic diversity of extant teleost species, it is likely that new efforts to sequence microbiomes from distantly related teleost species will reveal different assemblages to the ones so far reported. The bacterial communities present at different body sites (2), or under different conditions such as varying host developmental stages (3, 30), different diet regimes (30, 31) or following antibiotic treatment (32), have been sequenced. Importantly, fish also influence the bacterial composition of the tank water as evidenced by two different zebrafish studies (30, 33). Interindividual variation in microbial community composition has been reported in many different fish microbiome studies (2, 33, 34). Stephens et al. showed that in a group of zebrafish siblings raised in the same conditions, the gut microbiota still displays considerable interindividual variation. This variation can be explained at least in part by neutral processes of drift and dispersal (34). Additionally, ontogenic studies in zebrafish have shown that as the fish age, their gut microbial communities become increasingly different from that of the surrounding environment (30, 33). Whether these changes are also partially controlled by the host immune system is currently unknown. However, as discussed later, inter-host variability in the mucosal Ig repertoire may partially explain bacterial colonization in certain individuals but not others.

Based on sequencing studies from the gut and skin of turbot (35) and trout (2), respectively, it appears that fish are quite permissive in terms of mucosal tissue colonization. In other words, bacteria are not completely excluded from invading epidermal cells and goblet cells (2). This observation may have important consequences when investigating the interactions between the microbiota and the mucosal immune system of fish and further studies are required to understand the nature of this observed “permissiveness.”

Only a few comprehensive functional studies have provided a mechanistic view of the specific interactions that occur between bacterial symbionts and the fish immune system. Based on human microbiome studies, it is clear that microbiota regulates almost every aspect of the host physiology, including the immune response. Based on the seminal study on zebrafish gut responses to microbiota (36), it is tempting to speculate that most of the mechanisms underlying the control of immune systems by the mcirobiota in mammals may be conserved in teleosts. Undoubtedly, the great taxonomic diversity of fishes as well as their diverse physiological strategies and habitats likely results in very unique adaptations and coevolutionary processes not found in other vertebrate groups.

Whereas 16S rDNA next generation sequencing (NGS) has increased our understanding on bacterial communities of fish, future studies should investigate the archeal, fungal, and viral microbiota of fishes. Moreover, the inter-kingdom interactions between fish viriomes, mycobiomes, and bacteriomes remain unexplored. Similarly, functional studies of fish microbial community composition at different mucosal sites of the same individual require investigation.

## Germ-free Teleost Models: What have We Learned?

The development of germ-free zebrafish rearing techniques allowed researchers to compare the phenotype of zebrafish larvae, which develop in the absence of the microbiome with that of conventionally reared fish. Due to the laboratory research tools currently available, the majority of zebrafish studies have focused on the interactions between microbiota and the innate immune system. Germ-free zebrafish larvae have impaired neutrophil migration to injury sites (37), decreased larval resistance to viral infection (38), lack expression of innate immune genes, and altered gut epithelial cell turnover (39). Upon colonization with the natural microbiota, zebrafish larvae regain these immune functions. Thus, similar to mammals, teleost immune systems depend on the microbiota for stimulation to maintain a natural state of activity, which benefits the host.

Germ-free larvae can be used to conduct reassociation studies using the natural microbiota, single microbial species, or defined groups of microbes to determine direct effects of microbial presence on the immune system. These types of studies allow the identification of specific bacterial species and their interactions with the host immune system. Pioneer works on zebrafish revealed three main types of responses to specific bacterial colonization at the transcriptional level: innate immune responses, nutrient metabolism, and epithelial cell regeneration (36). Not all species are able to induce all three classes and bacterial products such as lippopolysaccharide (LPS) failed to elicit nutrient metabolism responses (36). Interestingly, germ-free zebrafish mono-associated with *Aeromonoas hydrophila* achieve higher induction of *serum amyloid a* expression and similar levels of *C3* expression as conventionalized larvae (39). The former result suggests that interactions between different members of the microbiota can serve to balance immunostimulatory effects of a single microbial member, while the latter result shows that single microbial species are sufficient to induce immunostimulatory effects. An elegant study by Rolig demonstrated that while in fish diassociated with *Vibrio* and *Shewanella, Vibrio* was the numerically dominant taxa, *Shewanella* presence significantly reduced neutrophil numbers compared to fish mono-associated with *Vibrio* (40). The latter challenges the assumption that the most abundant taxa exert the largest effects on host physiological and immune processes and suggests rarer species in the microbiota can exert potent effects on the immune system. Future studies on the extent of the immunomodulatory power of specific species within the microbiome, and whether these populations are sensitive to manipulation using antibiotics and probiotics will be highly impactful.

Some limitations of the germ-free zebrafish model are lack of known cell markers for immune cells, especially adaptive immune cells, which are not prominent during the early larval stages. Additionally, it is difficult to maintain the germ-free status of larvae past 7 dpf, as the larvae transitions from relying on yolk sac nutrients to eating food. While it is possible, though labor intensive, to maintain a zebrafish under germ-free conditions past this early life stage, no studies have been published using adult germ-free zebrafish. Conversely, a germ-free seabass model that incorporates germ-free feeding of live prey has recently been developed allowing for larvae to survive for at least 16 days post hatching, if not longer (9). Future refinement of the germ-free rearing technique in zebrafish and other teleost species, as well as identification of cell markers, production of reagents, and production of transgenic lines with reporters for or knockouts of important immune genes, will allow for a deeper understanding of the types of systemic immune responses that microbes are capable of inducing during development and adulthood in teleosts.

## Interactions Between Microbiota and the Teleost Mucosal Immune System

All fish mucosal sites are colonized by microbes, which interact with both the adaptive and innate immune system. Successful maintenance of immune homeostasis at these sites allow the microbiota to live as an extension of the teleost’s own physiology, providing essential functions in nutrient metabolism, maintenance of mucosal barriers, and protection from pathogens. In order to maintain this balance, microbes must either suppress or evade the host immune system, and the host immune system must be calibrated to prevent infection by opportunists, but remain tolerant to a natural number and diversity of microbes, which inhabit various niches in the mucosal microenvironment.

Both innate and adaptive immune pathways regulate bacterial colonization of mucosal surfaces (38, 41). With regards to innate immune pathways, MyD88 signaling appears to be critical (38). Activation of this pathway occurs due to the presence of MAMPs in the microbiota that exert innate immunomodulatory effects. For example, Bates and colleagues demonstrated, in 2007, in zebrafish that detection of LPS can induce intestinal alkaline phosphatase (IAP) expression *via* TLR4 detection and MyD88 signaling. In turn, IAP serves to detoxify LPS and maintain intestinal homeostasis. As mentioned earlier, germ-free teleost models have provided a detailed view of how microbial colonization triggers the transcription of different innate immune genes.

A sizeable fraction of microbes present at trout mucosal surfaces are coated by secreted IgT, IgM, and IgD as well as free SC (13, 14, 26, 27, 42). In mammals, it is generally thought that this coating is a form of immune exclusion, which allows the host to neutralize bacterial adhesion molecules to limit access to the host epithelium. Binding may be mediated by both antigen specific interactions between the Fab region of the antibody and non-specific interactions between glycosylated regions of the SC and antibodies and microbial surface receptors (43). Recently, *Flectobacillus major*-specific IgT titers were recorded in healthy hatchery rainbow trout gill and skin mucus. Interestingly, some fish also had *F. major*-specific IgM titers in plasma. Since both mucosal IgT and systemic IgM titers against this trout commensal strain were low, it was speculated that these antibodies are either natural antibodies or low-affinity cross-reactive antibodies that recognize common epitopes present in different commensal bacteria (44). Further studies should address whether exposure to commensals elicits compartmentalized Ig responses in mucosal and systemic sites similar to those elicited by pathogens.

Sepahi and Cordero also showed that *F. major*, an abundant microbe at trout mucosal surfaces, produces sphingolipids that induce IgT production in trout gill explants (44). *F. major*-derived sphingolipids injected intravenously into rainbow trout were capable of increasing the systemic IgT to IgM producing B cell ratio. Assuming other members of the microbial community are also producing an array of products, which can interact with immune system receptors, and acknowledging the co-evolution of the teleost immune system alongside the microbiota, it seems likely that the interplay between microbes, their products, and the immune system is highly complex and requires the balance between microbial and host molecules to have the tenacity to rebound to steady state conditions after stresses such as disease and environmental changes are placed on the fish. Future studies regarding the dynamics of how this balance is maintained depends on both the continued exploration of specific host–microbe interactions, as well as building a more accurate big-picture view of host–microbe interactions at mucosal surfaces.

Apart from interactions between B cell/Ig and microbiota, teleost T cells also shape the intestinal microbial composition (45). Adoptive transfer of T cells into Rag1-deficient zebrafish reduces the outgrowth of *Vibrio* sp. The *in vivo* mechanisms behind this inhibitory effect remain unexplored, but T lymphocytes exposed to the microbiota of Rag1-deficient zebrafish *in vitro* produced more IFNγ and TNFα compared to T lymphocytes exposed to the microbiota of wild-type zebrafish, suggesting T cell-mediated inflammatory responses may play a role in shaping the microbiome.

**Figure 1:**
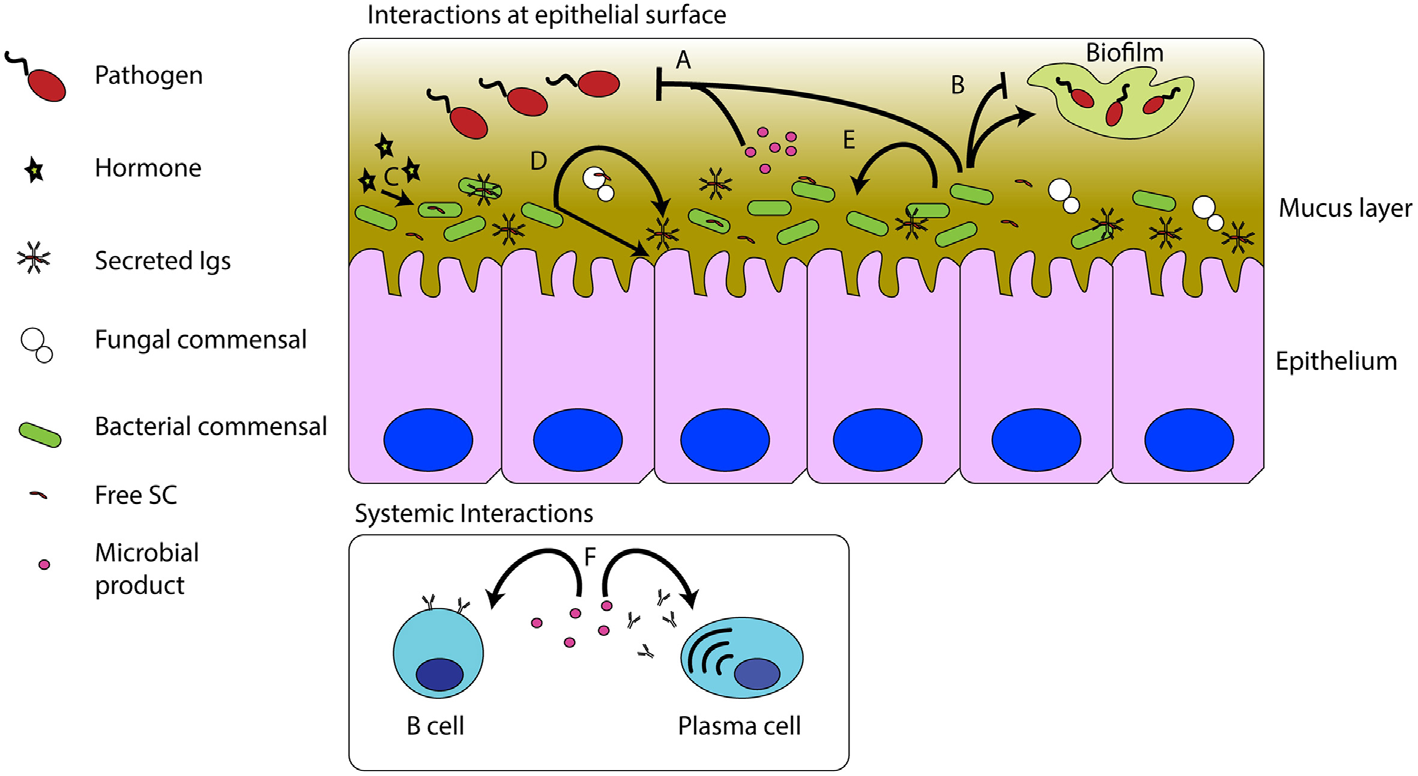
**Interactions between commensal microbes, pathogens, and the host immune system**. Commensal bacteria and their products can inhibit pathogenic infections **(A)**. Commensal microbes can promote or inhibit biofilm formation **(B)**. Host hormones, such as cortisol, can be sensed by, and have effects on commensals **(C)**. The presence of the microbiota stimulates S-Ig production and epithelial turnover **(D)**. Commensal microbes and their products can affect other commensal microbes **(E)**. Commensal products, such as sphingolipids, can modulate B cell numbers and antibody titers in mucosal and systemic compartments **(F)**.

Finally, it is worth highlighting the notion that microbiota contribute to the host’s array of immune defenses. Microbial products such as the aforementioned sphingolipids can affect the growth of other symbionts (44) or secrete molecules such as entericidin produced by *Enterobacter* sp., a trout commensal, which directly inhibits pathogen growth (46) in the same manner as host antimicrobial peptides would (Figure 1). On the other hand, when microbiota grows out of control, resident opportunists may favor colonization of pathogens, as demonstrated in the case of the commensal *Staphyloccocus warneri* and the pathogen *Vibrio anguillarum* (8).

## Interactions Between Microbiota and the Teleost Systemic Immune System

Despite the fact that multiple studies have shown that delivery of probiotic bacteria in fish diets can modulate teleost systemic immune responses and disease resistance (47–51), the mechanisms of this interaction remain unknown. As shown in Figure 1, fish commensal bacteria present in the gut mucosa can regulate certain systemic immune parameters. However, there is a clear knowledge gap concerning how these effects are achieved.

Possible indirect interactions between the microbiota and the teleost systemic immune system include production of metabolites such as carbohydrates, aminoacids, or lipids that can be uptaken by gut enterocytes and travel *via* the blood stream to systemic lymphoid tissues such as the HK or the spleen. For instance, PHB produced by *Bacteroides thuringensis* and delivered orally to Nile tilapia increases serum antibodies as well as innate immune parameters (52). However, how the PHB send this message to the systemic immune system is not understood.

Systemic delivery of commensal-derived metabolites has provided some useful insights into the possible mechanisms by which these bacterial products can regulate the fish immune system. For instance, intravenous (i.v.) delivery of *F. major* shingolipids is able to change IgM and IgT percentages in the HK (44). An overall increase in the proportion of lymphocytes in the HK 72 h after i.v. delivery suggests that this microbial product is able to stimulate B cell proliferation when it reaches systemic circulation. Thus, if the gill and skin of trout is able to extract sphingolipids from *F. major* or *F. major* itself is able to secrete these products and they can enter the bloodstream across the epithelial barriers, then systemic (HK) B cells could directly be controlled by symbiont products.

The contribution of commensal-derived aminoacids, CH, and lipids to the teleost host metabolic composition is unknown. Additionally, we do not know what metabolites commensal communities of fish are capable of producing and how they get secreted and absorbed. This lack of knowledge highlights the fact that implementation of microbiome intervention in aquaculture is still at its infancy.

## Interactions Between Microbiota and the Teleost Immune System during Stress or Disease

**Figure 2:**
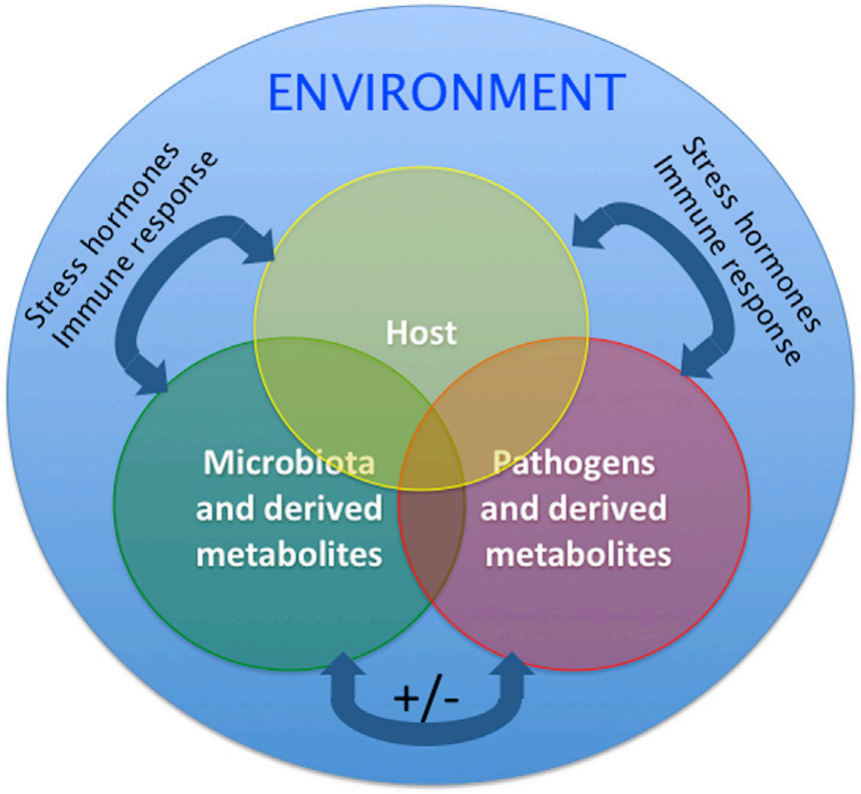
**Proposed model of the host–pathogen–microbiota interactions in aquatic animals such as teleost fish**. Two-way and three-way interactions among host, pathogen, and microbiota are possible and are overall affected by the environment. Interactions can be positive (synergistic) or negative (inhibitory). Additionally, the host microbial communities and host physiology modify the environment where the fish live. These interactions are, therefore, likely different in laboratory settings, aquaculture settings, and the wild. Homeostatic interactions form a delicate equilibrium. Under stress conditions, host, commensals, and pathogens can produce stress hormones that are molecularly conserved, will be released and alter the interactions of the triangle, likely resulting in decreased immune responses and outgrowth of opportunists and pathogens. Similarly, during the course of immune responses, release of immune molecules from host, commensals, and pathogens will shift the equilibrium of this triangle. The mechanisms by which teleost regain homeostasis following perturbations are largely unknown.

Microorganisms interact with each other to form resilient associations in humans (53). The application of microbial ecology concepts to the study of human microbiomes suggests that competitive rather than cooperative interactions between microbes foster the stability of the microbial communities (54). Spatiotemporal changes in the microbial composition of any given community take place during disturbances. In response to perturbations, functionally redundant members may become more abundant aiding in the preservation of community functionality. Environmental disturbances may differentially affect certain mucosal microenvironments. Thus, protected microenvironments could then act as reservoirs for recolonization of the disturbed regions (53). This theoretical framework and modeling has largely been applied to human gut microbiome studies as well as the assembly of the zebrafish microbiome during development. However, how fish microbial assemblages respond to disturbance is less well understood. It is worth noting that adapting this conceptual framework to fish likely needs to consider the greater influence of the environment on aquatic microbial communities compared to their terrestrial counterparts since water is a medium that highly supports microbial growth (Figure 2).

Overall, microbe–microbe interactions, host–microbiota interactions and host–pathogen interactions are complex and poorly understood (55). The dynamics of this triangle under homeostatic conditions require further investigation and may vary between teleost species. Additionally, although it is clear that any changes (i.e., altered microbiota or dysbiosis; altered host status such as stress of ongoing immune responses or altered pathogen loads) will result in loss of homeostasis and an unfavorable outcome for the host (Figure 2), the mechanisms that operate resilience and preservation of fish microbial communities remain poorly understood. Finally, it is very important to bear in mind that these interactions are likely different in a laboratory setting compared to the wild or a fish farm operation (12) as evidenced by the differences in the composition of zebrafish gut microbiomes from different laboratories (6).

A number of studies have shed some light onto the interactions between the microbiota and the teleost immune system during stress responses. For instance, transportation stress results in increased numbers of culturable skin mucus bacteria in rainbow trout. These changes in the skin microbiome were paralleled by change in gene expression of skin mucins, tight junction genes, and anti-inflammatory cytokines (56). Changes in bacterial numbers result in sharp differences in the host mucosal immune response. As suggested by a number of authors, the line between a symbiont and a pathogen is often a blurry one. Symbionts are generally defined as microorganisms that induce anti-inflammatory cytokine expression in the host, whereas pathogens induce pro-inflammatory responses (57, 58). However, even commensals will eventually trigger pro-inflammatory responses in the host if present at high enough numbers. It appears that this paradigm holds true in teleosts, since the commensal bacterium *S. warneri* induces anti-inflammatory cytokines in the skin of rainbow trout when present at low concentrations but pro-inflammatory cytokine expression is upregulated if high concentrations of the bacterium are achieved. Thus, stress-induced immunosupression likely allows local bacteria to overgrow.

In a separate study, hypoxic stress was shown to increase the relative abundance of putative pathogenic taxa such as *Psychrobacter, Steroidobacter, Pseudomonas, Acinetobacter, and Aeromonas* on trout skin (55). Stress has long been recognized as a key modulator of fish immunity with general immunosupressive effects (59, 60). Thus, not surprisingly, stress alters teleost microbiomes and results in dysbiosis. However, the mechanisms underlying stress-induced dysbiosis are unknown. Both direct (effects of hormones on the ability of certain bacterial taxa to grow) and indirect (inhibition of host immune responses by glucocorticoids) likely play a role.

We currently know very little about the impact of pathogens on the fish microbiota. One study evaluated the microbiota of wild tropical fish as well as their parasitic loads and found increased diversity of symbionts and lower presence of opportunists in fish that had greater parasitic burdens (61). This study, therefore, reveals a correlation between the presence of parasites and decreased presence of opportunistic pathogens. Whether the immune response of the host against the parasites is playing a role in decreasing opportunistic bacteria requires further investigation. Recently, the commercially important and devastating parasitic copepod *Lepeophtheirus salmonis* was shown to cause major changes in the Atlantic salmon skin microbiome by reducing the alpha diversity and causing destabilization of the microbial community composition (62).

Two reports have given some insights into bacterial diseases and the microbiome of fish (63). The first was conducted in farmed turbot and compared three different farms. This study, although it did not use deep-sequencing of the 16S rDNA, revealed that even healthy fish have a high abundance of bacteria present in internal organs such as the liver and kidney (63). However, the mucosal microbiomes of these fish were not studied and; therefore, it is unknown whether the internal organ microbial communities came from the healthy microbiota reservoir. More recently, the skin mucus microbiome of Atlantic salmon and smallmouth bass (*Micropterus dolomieu*) was studied using plate counts. Bacterial diversity was evaluated over time following natural *Aeromonas salmonicida* outbreaks in the fish farm (64). Despite the obvious limitation of the plate count method, authors concluded that microbial diversity decreased over time due to an over representation of *A. salmonicida* in the community. However, infection does not always result in losses in overall diversity of the microbiota, For instance, a recent study in laboratory seawater Atlantic salmon found no significant changes in the skin microbiome diversity (alpha diversity) of control and salmon alphavirus-infected fish due to high interindividual variability. However, experimentally infected salmon lost the majority of the proteobacteria and had increased abundances of opportunistic taxa (65). Thus, this study highlights a negative interaction between viral infection and the host–microbiota relationship. In both cases, the contribution of the host immune response to this outcome was not investigated.

## Concluding Remarks

Metazoans draw many benefits from the symbioses with prokaryotes. Unique partnerships have been selected through evolution in order to optimally exploit the metabolic capabilities of microorganisms. Teleost fish include >33,000 different extant species and; therefore, this diversity must be matched by a great diversity of selected microbial assemblages, which inhabit every fish mucosal barrier. Due to the conduciveness of the aquatic environment for microbial growth (66), it appears that minute changes in the host immune status can trigger states of dysbiosis. How teleost fish cope with these perturbations and how the microbial communities regain homeostasis is not fully understood. The complexity of the interactions between the environment, the teleost immune system, and the microbiota can now be dissected; thanks to NGS techniques, germ-free models, mono-association studies, and infection models. In the future, bacterial metagenomics and transcriptomic studies would be beneficial to advance our understanding of the functionality of fish microbiomes and their partnership with the fish immune system.

## Author Contributions

IS conceptually designed the paper and wrote the paper. CK wrote the paper and made figures.

## Conflict of Interest Statement

The authors declare that the research was conducted in the absence of any commercial or financial relationships that could be construed as a potential conflict of interest.
